# Efficacy and Mechanism of *Schisandra chinensis* Fructus Water Extract in Alzheimer’s Disease: Insights from Network Pharmacology and Validation in an Amyloid-β Infused Animal Model

**DOI:** 10.3390/nu16213751

**Published:** 2024-10-31

**Authors:** Hye-Jeong Yang, Ting Zhang, Min-Jung Kim, Haeng-Jeon Hur, Xuangao Wu, Dai-Ja Jang, Sunmin Park

**Affiliations:** 1 Food Functionality Research Division, Korea Food Research Institute, Wanju 55365, Republic of Korea; yhj@kfri.re.kr (H.-J.Y.); kmj@kfri.re.kr (M.-J.K.); mistltoe@kfri.re.kr (H.-J.H.); 2 Department of Food and Nutrition, Institute of Basic Science, Obesity/Diabetes Research Center, Hoseo University, Asan 31499, Republic of Korea; zhangting92925@gmail.com (T.Z.); niyani0@naver.com (X.W.); 3 Department of Bioconvergence, Hoseo University, Asan 31499, Republic of Korea

**Keywords:** *Schisandra chinensis* Fructus, network pharmacology, prostaglandin-endoperoxide synthase 2, acetylcholinesterase, Alzheimer’s disease

## Abstract

Background/Objectives: *Schisandra chinensis* Fructus (SCF) is a traditional medicinal herb containing lignans that improves glucose metabolism by mitigating insulin resistance. We aimed to investigate the therapeutic potential and action mechanism of SCF for Alzheimer’s disease (AD) using a network pharmacology analysis, followed by experimental validation in an AD rat model. Methods: The biological activities of SCF’s bioactive compounds were assessed through a network pharmacology analysis. An AD rat model was generated by infusing amyloid-β peptide (Aβ) (25–35) into the hippocampus to induce Aβ accumulation. The AD rats were fed either 0.5% dextrin (AD-Con) or 0.5% SCF (AD-SCF group) in a high-fat diet for seven weeks. The rats in the normal/control group received an Aβ (35–25) infusion (no Aβ deposition) and were fed a control diet (Normal-C). Aβ deposition, memory function, inflammation, and glucose/lipid metabolism were evaluated. Results: The network analysis revealed significant intersections between AD-related targets and bioactive SCF compounds, like gomisin A, schisandrin, and longikaurin A. Key AD genes prostaglandin-endoperoxide synthase-2 (*PTGS2*, cyclooxygenase-2) and acetylcholinesterase (*AChE*) were linked to SCF compounds. In the rats with AD induced by bilaterally infusing amyloid-β (25–35) into the hippocampus, the 0.5% SCF intake mitigated hippocampal amyloid-β deposition, neuroinflammation, memory deficits, and dysregulated glucose and lipid metabolism versus the AD controls. SCF reduced hippocampal AChE activity, inflammatory cytokine expression related to *PTGS2*, and malondialdehyde contents and preserved neuronal cell survival-related factors such as brain-derived neurotrophic factor and ciliary neurotrophic factor similar to normal rats. The neuroprotective effects validated the network analysis findings. Conclusions: SCF could be a potential AD therapeutic agent by activating the parasympathetic nervous system to reduce hippocampal oxidative stress and inflammation, warranting further clinical investigations of its efficacy.

## 1. Introduction

Alzheimer’s disease (AD) is a neurodegenerative disorder marked by amyloid-β (Aβ) plaques and tau-containing neurofibrillary tangles, leading to progressive cognitive decline and behavioral changes [1]. The pathogenesis of AD involves a critical interplay between cholinergic system dysfunction and metabolic disorders like type 2 diabetes [2]. Cholinergic neurons, essential for both cognitive function and glucose metabolism [3], undergo significant degeneration in AD, resulting in reduced acetylcholine levels [2]. This condition is exacerbated by elevated acetylcholinesterase (AChE) activity [4], while insulin resistance further disrupts acetylcholine synthesis and increases AChE activity, creating a feedback loop that accelerates cognitive decline [5].

As populations around the world age, the prevalence of AD is expected to rise, making it a growing public health concern. AD etiology involves a multifactorial interplay between genetic predisposition and lifestyle factors. The primary genetic mutations associated with AD include amyloid-β precursor protein (*APP*), presenilin *(PSEN)-1*, *AChE*, and *PSEN2*. The apolipoprotein E (*APOE*) genotype, especially the ApoE ε4 allele, represents a genetic risk factor for AD. Lifestyle factors include physical inactivity, poor diet, lack of cognitive stimulation, social isolation, and chronic stress [6]. The accumulation of abnormal protein aggregates, particularly Aβ and tau, contributes to neuronal dysfunction and cognitive decline [1]. Additionally, the activation of microglia, the immune cells in the brain, and the release of inflammatory molecules contribute to neuronal damage and degeneration [7]. Vascular risk factors, such as hypertension, diabetes, obesity, and hypercholesterolemia, can impair blood flow to the brain and increase Aβ deposition in the hippocampus, increasing one’s susceptibility to neurodegeneration [8].

Despite decades of research, effective treatments for AD remain elusive, highlighting the need for innovative therapeutic approaches [9]. AD is a long-term, chronic disease, and the development of safe and effective long-term therapies is crucial. Herbal medicines, which have been traditionally used for a long time, offer a promising avenue for developing novel and more effective therapeutic strategies [10]. *Schisandra chinensis* Fructus (SCF), a widely used traditional Chinese medicine, has garnered attention for its potential to prevent and manage metabolic diseases [11,12]. The neuroprotective and cognition-enhancing effects of SCF are based on its multiple mechanisms of action, including its potent antioxidant and anti-inflammatory properties [12]. However, the specific mechanisms underlying the therapeutic efficacy of SCF in AD have yet to be fully elucidated.

Network pharmacology, a systems biology-based approach, offers a promising framework for understanding the complex interactions between drugs, targets, and diseases [13]. By elucidating these intricate relationships, network pharmacology can provide valuable insights for developing novel treatment strategies [14]. Network pharmacology can be particularly valuable in unraveling the multitarget and multi-pathway effects of natural products, such as SCF, and revealing their potential therapeutic mechanisms in specific diseases [13]. This system-level understanding of how the bioactive compounds in SCF interact with the key targets and pathways involved in AD pathology can offer crucial insights to guide the exploration of SCF as a novel, multifaceted therapeutic approach for this devastating neurodegenerative disorder.

This study aimed to evaluate the therapeutic potential of SCF water extract in AD through the dual approach of a network pharmacology analysis and experimental validation. Our primary objectives were as follows: (1) to identify and characterize the bioactive compounds in SCF and their molecular targets in AD pathology through a network pharmacology analysis; and (2) to validate the therapeutic effects of SCF water extract in an Aβ-induced AD rat model. Using network pharmacology, we systematically analyzed the bioactive compounds in SCF and their interactions with AD-related molecular targets to elucidate the underlying mechanisms of action. These predicted mechanisms were then experimentally validated using an AD rat model, in which AD was induced by Aβ infusion into the hippocampus. This integrated approach, combining computational predictions with experimental validation, allowed us to comprehensively evaluate SCF’s neuroprotective effects and its potential as a therapeutic intervention for AD.

## 2. Materials and Methods

### 2.1. Identification of Bioactive Components in SCF and Associated Genes

Bioactive components of *Schisandra chinensis* Fructus (SCF) were initially identified through a comprehensive database search, including the Traditional Chinese Medicine Systems Pharmacology Database and Analysis Platform (TCMSP), PubChem, ChEMBL, and FooDB using search terms such as “*Schisandra chinensis* Fructus”, “Schisandra”, “Wu Wei Zi”, and “Omija” [15]. The search yielded 130 compounds. The compounds were filtered using two established pharmacokinetic criteria: oral bioavailability (OB) ≥ 30% and drug-likeness (DL) ≥ 0.18 [14], resulting in 8 bioactive components that met both thresholds. The target genes of these selected SCF compounds and their encoded proteins were then investigated using TCMSP, PubChem, ChEMBL, GeneCards^®^, and OMIM^®^. Separately, AD-related genes were identified through database searches across OMIM^®^, DrugBank, GeneCards^®^, and DisGeNET, selecting genes with relevance scores ≥ 30.

The targets of the active ingredients of SCF were integrated with the disease targets related to AD, and all targets were converted into a unified UNIPROT ID format. The complete information was obtained through the Uniport database (https://www.uniprot.org/, accessed on 6 March 2024), and the VENNY 2.1 tool (https://bioinfogp.cnb.csic.es/tools/venny/index.html, accessed on 6 March 2024) was used for analysis to screen out the potential common targets of the active ingredients of SCF and AD.

### 2.2. Constructing the “Drug–Component–Disease–Target” Network

To systematically identify potential therapeutic targets, we analyzed the intersection between the AD-related gene set and the SCF bioactive component target genes using a Venn diagram analysis tool, which provided a visual representation of the overlapping genes between these two sets. To explore the potential therapeutic targets of the SCF components to treat AD, a comprehensive “drug-component-disease-target” network was constructed using the Cytoscape 3.8.2 software [14]. This network contained nodes representing the target genes associated with the bioactive compounds of SCF and AD. The edges connecting the nodes depicted the interactions between the plant components, their targets, and the disease. The principle of node connection is to use edges to represent the relationship between active ingredients and target genes and between AD and target genes. By constructing a network topology, the multi-component, multi-target, and multi-pathway mechanism of action of SCF in AD treatment can be intuitively displayed, which is helpful for further analysis.

### 2.3. Screening Key Targets Based on Network Analysis and Protein–Protein Interactions

Key therapeutic targets were screened using the “average degree” criterion, in which genes with more connections (higher degrees) in the network are assumed to be more critical for disease pathology and treatment. Targets with degrees higher than the average were identified as candidates likely linked to AD and influenced by SCF bioactive components.

This initial network analysis was validated using the STRING database for protein–protein interactions in *Homo sapiens* (confidence ≥ 0.9) [14]. STRING data were imported into Cytoscape version 3.8.2. Its “Network Analyzer” function identified key target proteins whose degrees exceeded the average node degree in the STRING-derived network. Integrating the original network and STRING-based analysis established a robust set of critical therapeutic targets for SCF compounds in AD treatment. A network approach determined the drug–disease intersection targets by comparing SCF component targets with known AD-related targets. These intersection targets underwent pathway and functional enrichment analyses.

### 2.4. Pathway and Functional Enrichment Analyses

Two widely used bioinformatics resources, the Kyoto Encyclopedia of Genes and Genomes (KEGG) and Gene Ontology (GO), were employed to gain insights into the biological pathways and functions associated with the identified targets [14]. The KEGG enrichment analysis utilized the KEGG database, a comprehensive repository of manually curated pathways representing molecular interactions and reaction networks [14]. The GO enrichment analysis leveraged the Bioconductor database, a software package providing tools for analyzing and interpreting high-throughput genomic data.

These analyses identified statistically significant pathways and functional categories among proteins encoded by target genes associated with SCF compounds and AD. Significance was assessed using *p*-values < 0.05 for GO and q-values < 0.05 for KEGG enrichment. The analyses provided insights into potential mechanisms by which SCF components may exert therapeutic effects in AD.

### 2.5. SCF Production

Dried SCF (#HSS-19) from Mungyeong-si, Republic of Korea, underwent two 5 h water extractions at 50 °C, filtration, centrifugation at 450× *g*, and lyophilization. The dried extract was dissolved in methanol, and lignans were quantified using HPLC (Waters Co., Milford, MA, USA) with a Gemini C18 column (150 × 2.0 mm; 5 µm; Phenomenex, Torrance, CA, USA), detected at 280 nm, with a 0.8 mL/min flow rate and a 55 min acetonitrile gradient. Reference compounds (gomisin A and C; schisandrin A, B, and C) with >98% purity were from Sigma-Aldrich Co. (St. Louis, MO, USA). Lignan retention times and contents are shown in Figure S1.

### 2.6. AD-Induced Animal Model and Experimental Design

**Figure 1:**
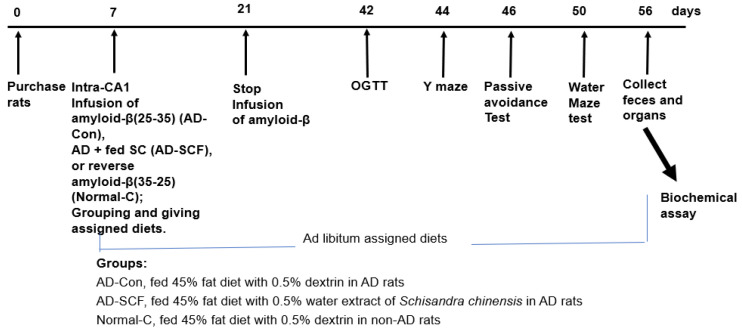
Experimental design.

In vivo experiment was approved by the Animal Care and Use Review Committee at Hoseo University, Republic of Korea (HSIACUC-20021). The experimental design is presented in Figure 1. Male Sprague Dawley rats (192 ± 30 g) were housed individually under controlled conditions (23 °C; 12 h light/dark cycle).

The experimental design is presented in Figure 1. On the seventh day after the adjustments were made in the animal facility, rats were anesthetized with the intraperitoneal injection of ketamine and xylazine (100 mg and 10 mg/kg body weight), and a cannula was implanted in the bilateral CA1 region of the hippocampus (lateral: −3.3 mm from bregma; posterior: 2.0 mm from midline; ventral: −2.5 mm from dura) to infuse Aβ (25–35) (AD group) or Aβ (35–25) (Normal-C group) at 3.6 nmol/day for 14 days using an osmotic pump (Alzet Osmotic Pump Company) [16]. The Aβ (25–35) fragment is a portion of the larger Aβ peptide that can be transported across the blood–brain barrier, resulting in neurotoxicity, as observed in in vivo models.

The AD group (n = 20) was randomly divided into AD-Con (0.5% dextrin diet) and AD-SCF (0.5% SCF diet) groups (n = 10 each). Normal-C (n = 10) received the AD-Con diet. All had ad libitum water/food access for 7 weeks and were fed a high-fat diet, inducing insulin resistance. The high-fat diet contained 37 energy percent (En%) carbohydrates, 20 En% protein, and 43 En% fats from starch plus sugar, casein, and lard, respectively. Dextrin or SCF (0.5%) was added to the diet. After overnight fasting, serum glucose, food intake, and body weight were measured weekly.

On day 42, an oral glucose tolerance test (OGTT) was performed by orally giving them 2 g/kg glucose and measuring serum glucose (every 10 min up to 90 min and then at 120 min) and insulin (20, 40, 60, and 90 min). The homeostasis model assessment for insulin resistance index (HOMA-IR) was calculated using the following formula: Serum insulin (μU) × serum glucose (mM)/22.5.

### 2.7. Y-Maze, Passive Avoidance, and Water Maze Tests

On day 44, rats were placed in one arm of a Y-shaped maze with three arms that were 50.5 cm long, 20 cm wide, and 20 cm high, and their movements were monitored for 8 min [16]. Correct alternations (consecutive entries into all 3 arms) were recorded, and % spontaneous alternation was calculated as correct alternations/total arm entries.

On day 46, the passive avoidance test was conducted to assess short-term memory using a shuttle box (light and dark chambers). During the acquisition trials, rats received 75 V, 0.2 mA, and 50 Hz foot stimulation for 5 sec upon entering the dark chamber. 24 h later, the same procedure was conducted without stimulation as a retention trial [17]. The latencies to enter the dark chamber (max 600 s) were recorded, with longer latencies indicating better memory retention.

The water maze setup consisted of a circular pool with specific dimensions (diameter: 150 cm, height: 50 cm), filled with water maintained at a temperature of 22 ± 1 °C to a depth of 30 cm. Over five consecutive days, rats were subjected to three trials each day. In each trial, rats were placed at one of four predetermined starting positions, facing the pool wall, and tasked with finding a hidden platform within 60 s. The time to find the platform (escape latency) and the swimming path were meticulously recorded using a video tracking system. On the fifth day, a probe trial was conducted wherein the platform was removed from the pool. Rats were given 300 s to swim freely, and their time spent in the quadrant where the platform was previously located was measured to evaluate long-term spatial memory retention.

On the following day of the last water maze trial, blood, feces, and organs were collected from the rats, and they were anesthetized with ketamine and xylazine at a dose of 100 and 10 mg/kg bw. The brains of six randomly selected rats were immersed overnight in a 20% sucrose solution and then frozen at −20 °C. The brains of the remaining four rats were dissected to isolate the hippocampus, which was divided into two parts. One portion was lysed with RIPA buffer (composition: 50 mM Tris-HCl, 150 mM NaCl, 1.0% (*v*/*v*) IGEPAL CA-630, 0.5% (*w*/*v*) sodium deoxycholate, 1.0 mM EDTA, 0.1% (*w*/*v*) SDS, and 0.01% (*w*/*v*) sodium azide; pH 7.4) for protein extraction, while the other portion was lysed with TRIzol (Invitrogen, Rockville, MD, USA) for total RNA extraction. After centrifugation of the lysed cells with RIPA buffer, the supernatants were collected to measure the triglyceride peroxide and lipid contents with lipid peroxide and triglyceride spectrophotometric kits. The supernatants were digested with α-amyloglucosidase (Sigma, St. Louise, MO, USA), and glucose contents were measured with a glucose spectrophotometric kit to calculate glycogen contents (AM201; Asan Pharmaceuticals, Seoul, Republic of Korea). Acetylcholinesterase (AChE) activity and PTSG-2 in the hippocampal lysate were also assessed using the rat AChE (E-EL-R0355) and rat PTSG-2 ELISA kits (E-EL-R0792; Elabscience, Houston, TX, USA), respectively. Tumor necrosis factor-α (TNF-α) and interleukin (IL)-1β in the serum and hippocampal lysates were measured with rat TNF-α (KRC3011) and IL-1β (BMS630) ELISA kits (Invitrogen, Carlsbad, CA, USA), respectively. Serum insulin concentration was measured with a rat ultrasensitive insulin ELISA kit (90060; Crystal Chem, Elk Grove Village, IL, USA).

### 2.8. Quantitative Real-Time PCR

Total RNA was extracted from the other portion of the hippocampal tissues of 4 rats per group using TRIzol (Invitrogen, Rockville, MD, USA). cDNA was synthesized using reverse transcriptase (18090010) and Taq polymerase (12574018; Invitrogen, Rockville, MD, USA). Real-time PCR was performed using SYBR Green and primers for ciliary neurotrophic factor (CNTF), brain-derived neurotrophic factor (BDNF), TNF-α, IL-1β, IL-6, PTGS-2, and β-actin (reference gene) on a Bio-Rad PCR machine (Hercules, CA, USA). The primer sequences for CNTF, BDNF, TNF-α, IL-1β, IL-6, PTGS-2, and β-actin were obtained from a previous study [18]. The comparative CT method [19] was used for gene expression calculation.

### 2.9. Brain Immunohistochemistry Analysis

For cryoprotection, the fixed brain tissues were immersed in a 20% sucrose solution. The cryoprotected brains were frozen and serially sectioned into 30 μm coronal sections using a cryostat (Leica, Wetzlar, Germany). Immunohistochemistry was performed on the brain sections using a specific antibody against amyloid-β [20,21]. The extent of amyloid-β deposition was quantified as the percentage of amyloid-β-positive cells in the hippocampal area [21].

### 2.10. Statistical Analysis

SAS version 7 (SAS Institute in Cary, NC, USA) was used for the statistical analysis. It was determined that a sample size of 10 subjects per group, calculated using the G power program with a power of 0.85 and an effect size of 0.50, would provide sufficient power to evaluate the main effects. The results are presented as means ± standard deviations (SDs). For normally distributed variables, univariate analysis was employed. One-way analysis of variance (ANOVA) was used to compare the statistical differences among the groups. In cases in which one-way ANOVA revealed a significant difference between the groups, Tukey’s test was applied for multiple group comparisons. *p*-values < 0.05 were statistically significant.

## 3. Results

### 3.1. Results of the Network Analysis

#### 3.1.1. Identification of SCF Active Compounds and Their Targets

Among the 130 compounds identified in SCF from the TCMSP database, eight bioactive components met the pharmacokinetic criteria (oral bioavailability ≥ 30% and drug-likeness ≥ 0.18): longikaurin A, deoxyharringtonine, angeloylgomisin O, schizandrer B, gomisin A, gomisin G, gomisin R, and schisandrin C. An analysis across multiple databases (TCMSP, PubChem, ChEMBL, GeneCards^®^, and OMIM^®^) identified 30 unique potential targets for these compounds (Supplementary Table S1).

#### 3.1.2. Identification of AD-Related Target

The search for “Alzheimer’s disease” yielded varying numbers of disease targets from each database, with 384 from OMIM^®^, 658 from DrugBank, 2943 from GeneCards^®^, and 826 from DisGeNET. After combining the results from all four databases and eliminating duplicate entries, a total of 2338 potential targets related to AD were identified for further analysis.

#### 3.1.3. Drug–Ingredient–Disease–Target Network Analysis

**Figure 2:**
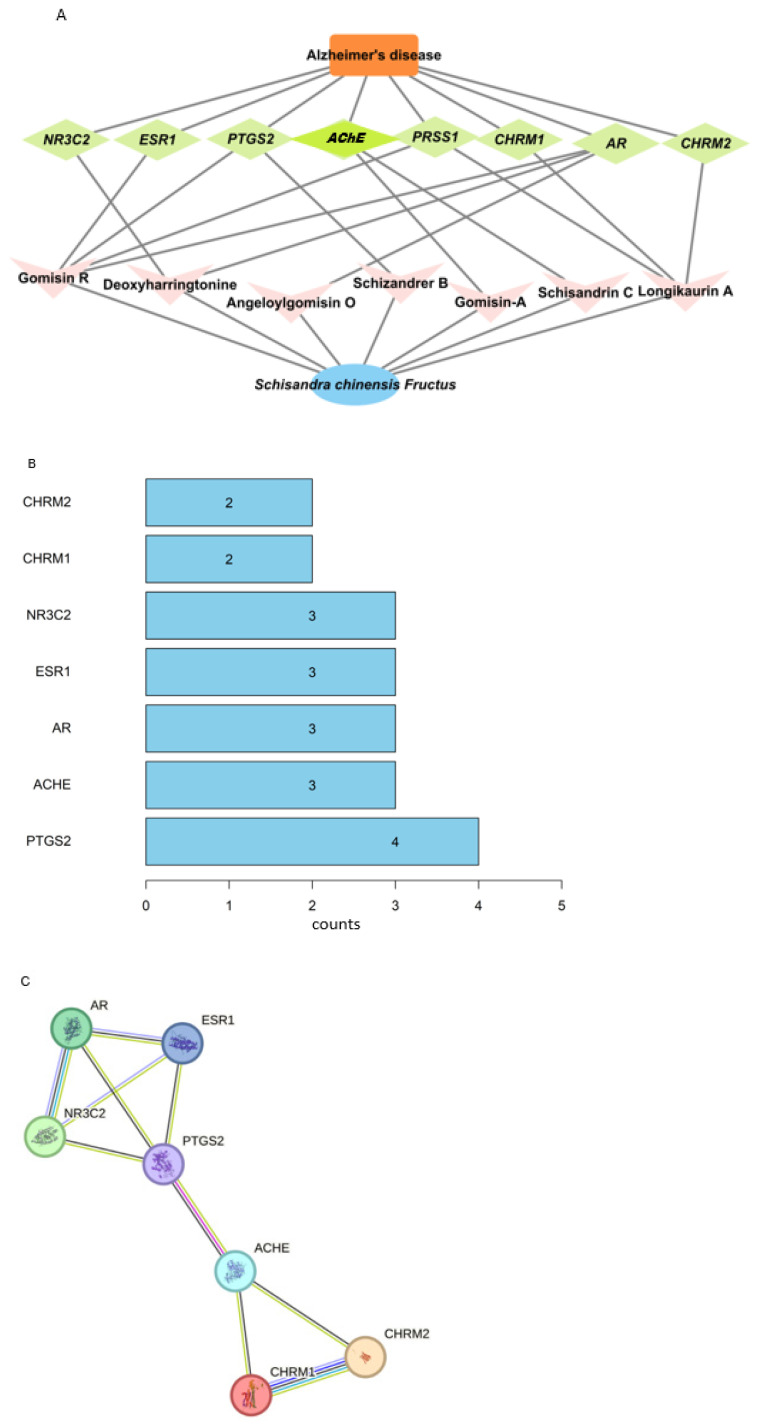
Network pharmacology results between the bioactive compounds of *Schisandra chinensis* Fructus (SCF) and the molecular targets of Alzheimer’s disease (AD). (**A**) Network between the bioactive compounds of SCF and the molecular targets of AD. The orange square, green eclipse, pink V, and blue eclipse represent AD, molecular targets, SCF bioactive compounds, and SCF herbs, respectively. The lines indicate the interaction between the SCF bioactive compounds and the molecular targets of AD. Network pharmacology analysis was conducted using Cytoscape-3.8.2, National Resource for Network Biology, National Institute of Health, Bethesda, MD, USA (http://www.cytoscape.org). (**B**) The interaction counts with AD and SCF ingredients with molecular targets. (**C**) Interaction of molecular targets.

The Venn diagram identified eight common targets between SCF and AD (Supplementary Figure S1). The drug-ingredient-disease-target network analysis revealed the complex interactions between SCF bioactive components and Alzheimer’s disease (AD) molecular targets (Figure 2A). In this network visualization, the pink diamonds represent SCF bioactive components, the green diamonds indicate molecular target genes, the blue ellipse shows SCF, and the orange square represents AD. The interconnecting lines demonstrate direct associations between these elements, which were established through database mining and evidence from the literature. Our analysis revealed varying degrees of connectivity among the bioactive components: gomisin R and longikaurin A showed the highest number of connections to AD-related molecular targets, suggesting their potential importance as key therapeutic compounds. Notably, the *AChE* gene, a crucial target in AD pathology, showed specific connections to gomisin A and schisandrin C, indicating these compounds may influence cholinergic signaling pathways. Based on the network topology and connection patterns, we identified gomisin A, gomisin R, longikaurin A, and schisandrin C as the most promising bioactive components for AD therapy, due to their multiple interactions with disease-relevant molecular targets.

#### 3.1.4. Protein–Protein Interaction (PPI) Network and Pathway Analysis

The PPI network analysis showed that seven genes interacted with each other (Figure 2B). *PTGS2* had four interactions, and nuclear receptor subfamily 3 group c member 2 (*NR3C2*), estrogen receptor alpha (*ESR1*), androgen receptor (*AR*), and *AChE* showed three interactions with other genes (Figure 2B). *PTGS2* interacted with *AR, ESR, NR3C2*, and *AChE*, while *AChE* interacted with cholinergic receptor muscarinic 1 (*CHRM1), CHRM2,* and *PTGS2*. The interaction suggested that *PTGS2* and *AChE* were the primary AD-associated genes that could be influenced by treatment with the bioactive components of SCF (Figure 2C).

**Figure 3:**
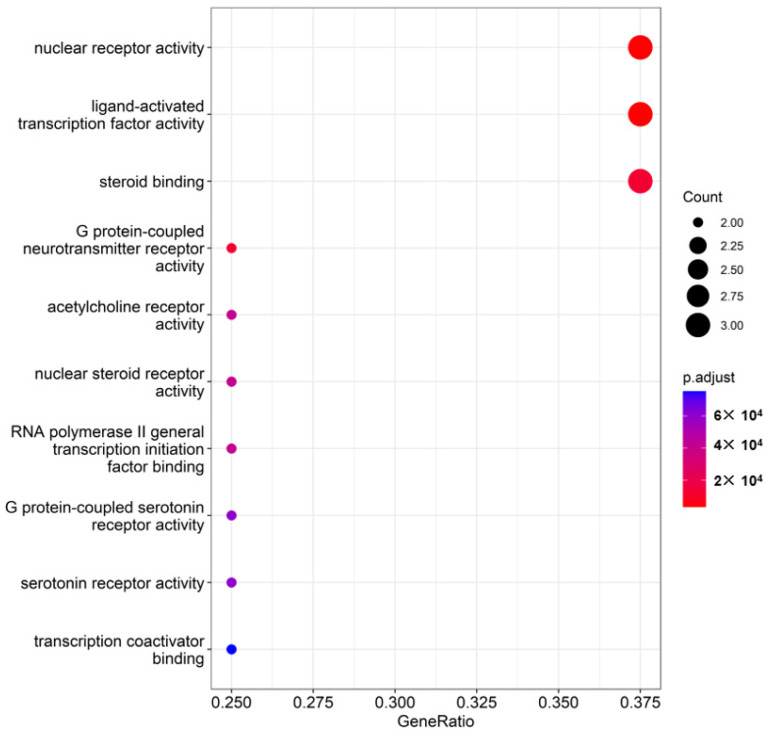
Gene ontology (GO) enrichment analysis between Alzheimer’s disease (AD) and *Schisandra chinensis* Fructus (SCF) compounds.

In the GO enrichment analysis, nuclear receptor activity, ligand-activated transcription factor activity, and steroid binding showed 3.0 counts (*p* = 2 × 10^−4^). G-protein-coupled neurotransmitter receptor activity, acetylcholine receptor activity, nuclear steroid receptor activity, RNA polymerase II general transcription initiation factor binding, G-protein-coupled serotonin receptor activity, serotonin receptor activity, and transcription coactivator binding had 2.0 counts (Figure 3; *p* = 4–6 × 10^−4^). These results showed that SCF primarily impacted the nuclear receptor activity with *ERS-1*, *AR*, and *NR3C2* and the G-protein-coupled neurotransmitter receptor activity involving *AChE, CHRM1*, and *CHRM2*. *PTGS2* was involved in inflammation, and it acted as a connector between neurotransmitter receptor activity and G-protein-coupled neurotransmitter receptors, modulating memory function and neuronal cell survival.

The KEGG pathway analysis identified the cholinergic synapse as the sole significantly enriched pathway (*p* = 0.0048; Figure S2A). This finding aligns with our network analysis results, which identify AChE as a key target and support SCF’s potential role in modulating cholinergic neurotransmission, which is disrupted in AD pathology. The cholinergic synapse mechanism suggested SCF’s potential role in modulating acetylcholine signaling through regulating synthesis, release, and degradation processes (Figure S2B). In detail, these included acetylcholine synthesis by CHAT in presynaptic terminals, vesicular transport via SLC18A3, calcium-dependent release into the synaptic cleft, and binding to postsynaptic nicotinic/muscarinic receptors. This signaling cascade modulates synaptic plasticity and neuronal survival through calcium and MAPK pathways, with signal termination occurring via AChE-mediated acetylcholine hydrolysis.

### 3.2. Results of SCF’s Anti-AD Effect in AD-Induced Rats

#### 3.2.1. Gomisin and Schisandrin Content in SCF

Contents of 7.04 ± 0.12 µg gomisin A, 1.03 ± 0.09 µg gomisin C, 8.82 ± 0.16 µg schisandrin A, 2.52 ± 0.09 µg schisandrin B, and 1.36 ± 0.07 µg schisandrin C per 1 g dried SCF were detected (Figure S3).

#### 3.2.2. Changes in Body Weight and Glucose Metabolism

Body weight in the fifth week and weight gain during the experimental period did not differ among the groups (Table 1). However, epididymal fat and retroperitoneal fat contents, representing visceral fat mass, were higher in the AD-Con than in the Normal-C group and similar in the AD-SCF and Normal-C groups. Fasting serum glucose concentrations were higher in the AD-Con than in the Normal-C group and lower in the AD-SCF than in the AD-Con group (Table 1). Fasting serum insulin concentrations were higher in the AD-Con group than in the Normal-C group and were not significantly different between the AD-Con and AD-SCF groups. HOMA-IR, the insulin resistance index, was higher in the Normal-C, AD-SCF, and AD-Con groups, in ascending order. The serum TNF-α concentration showed a pattern similar to that of HOMA-IR (Table 1).

**Figure 4:**
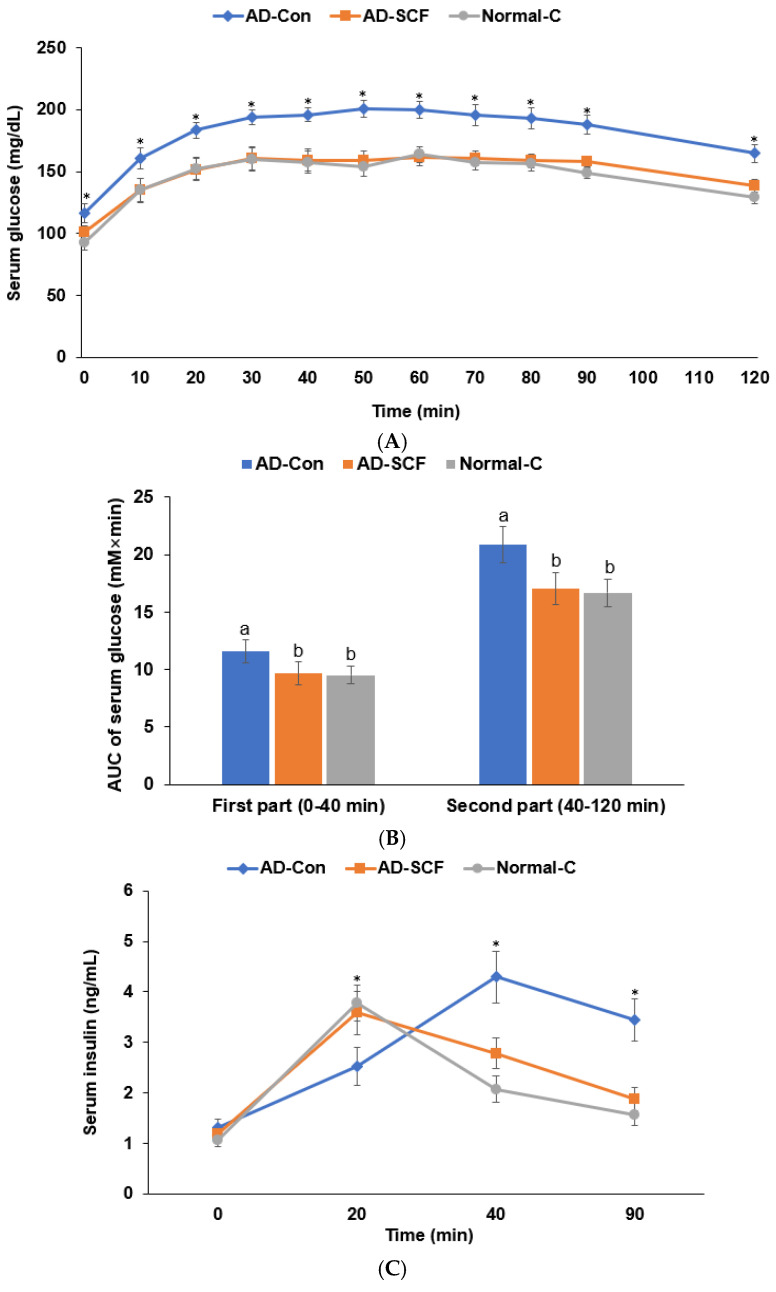

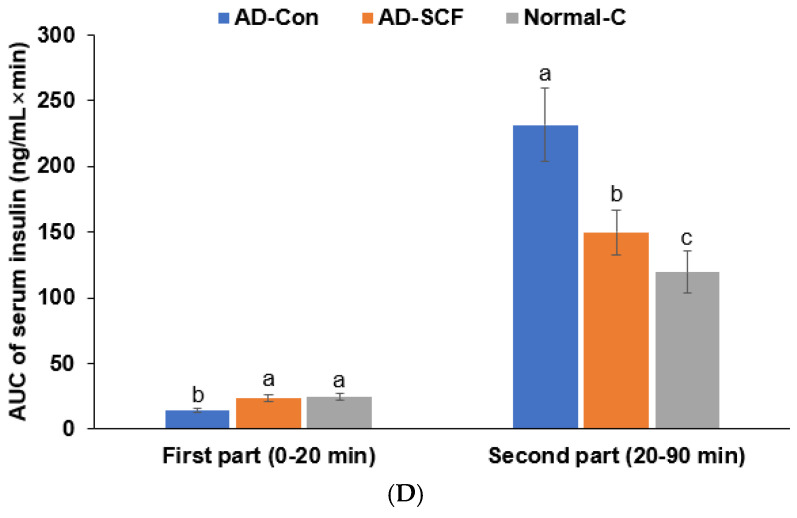
Serum glucose and insulin concentrations during oral glucose tolerance tests (OGTTs). (**A**) Changes in serum glucose concentrations 120 min after the oral administration of 2 g glucose/kg body weight. (**B**) Area under the curve (AUC) of serum glucose concentrations during the OGTT. (**C**) Changes in serum insulin concentrations during the OGTT. (**D**) Area under the curve (AUC) of serum insulin concentrations during the OGTT. Each bar and error bar represents means ± standard deviation (n = 10). The Aβ (25-35)-infused AD model rats were fed high-fat diets with 0.5% dextrin (AD-Con) and 0.5% *Schisandra chinensis* Fructus water extracts (SCF; AD-SCF) for 7 weeks. The Aβ (35-25)-infused rats were fed high-fat diets with 0.5% dextrin (Normal-C). * Significantly different among the groups at each time point at *p* < 0.05. ^a,b,c^ Different letters on the bars indicate a significant difference among the groups according to Tukey’s test at *p* < 0.05.

On day 42, glucose was orally loaded in proportion to body weight (2 g/kg BW). The serum glucose concentrations were elevated until 40–60 min and then slowly decreased until 80 min in all the groups (Figure 4A). However, the serum glucose concentrations at 40 min were higher in the AD-Con group than in the other groups, and they decreased more slowly in the AD-Con group. Serum glucose concentrations were higher at 120 min in the AD-Con group than in the AD-SCF and Normal-C groups (Figure 4A). Serum glucose concentrations were similar in the AD-SCF and Normal-C groups throughout the OGTT. The area under the curve (AUC) of the serum glucose concentrations from 0 to 40 min (the first part) and from 40 to 120 min (the second part) during the OGTT were higher in the AD-Con group than in the AD-SCF and the Normal-C groups (Figure 4B). The AUC of the first and second parts was similar in the AD-SCF and the Normal-C groups (Figure 4B). The serum insulin concentration was the highest at 40 min in the AD-Con group, while it was the highest at 20 min in the AD-SCF and Normal-C groups (Figure 4C). The serum insulin concentration was higher at 40 and 120 min in the AD-Con group than that in the other groups (Figure 4C). The AUC of serum insulin from 0 to 20 min was lower in the AD-Con group than in the AD-SCF and Normal-C groups, and from 20 to 90 min, the AUC of serum insulin was highest in the AD-Con group, followed by the AD-SCF and Normal-C groups (Figure 4D).

#### 3.2.3. Lipid Parameters in the Blood and Hippocampus

The serum triglyceride concentrations were higher in the AD-Con group than in the Normal-C group and were similar between the AD-SCF and Normal-CON groups (Table 2). This suggested SCF’s role in suppressing the increase in triglycerides. The serum cholesterol concentrations also showed a trend similar to the serum triglycerides, but there was no significant difference between the AD-Con and AD-SCF groups. The serum lipid peroxide concentration increased in the AD-Con group compared to the Normal-C group, and the addition of SCF retained this increment (Table 2). The hippocampal triglyceride and cholesterol content were higher in the AD-Con group than in the Normal-C group, and they were similar between the AD-SCF and the Normal-C groups (Table 2). Furthermore, triglyceride and cholesterol deposition in the hippocampus was higher in the AD-Con group than in the Normal-C group, and the levels in the AD-SCF and Normal-C groups were similar. Conversely, hippocampal glycogen deposition was lower in the AD-Con group than in the Normal-C group and comparable to that in the AD-SCF and Normal-C groups (Table 2).

#### 3.2.4. Aβ Deposition and Memory Deficit

**Figure 5:**
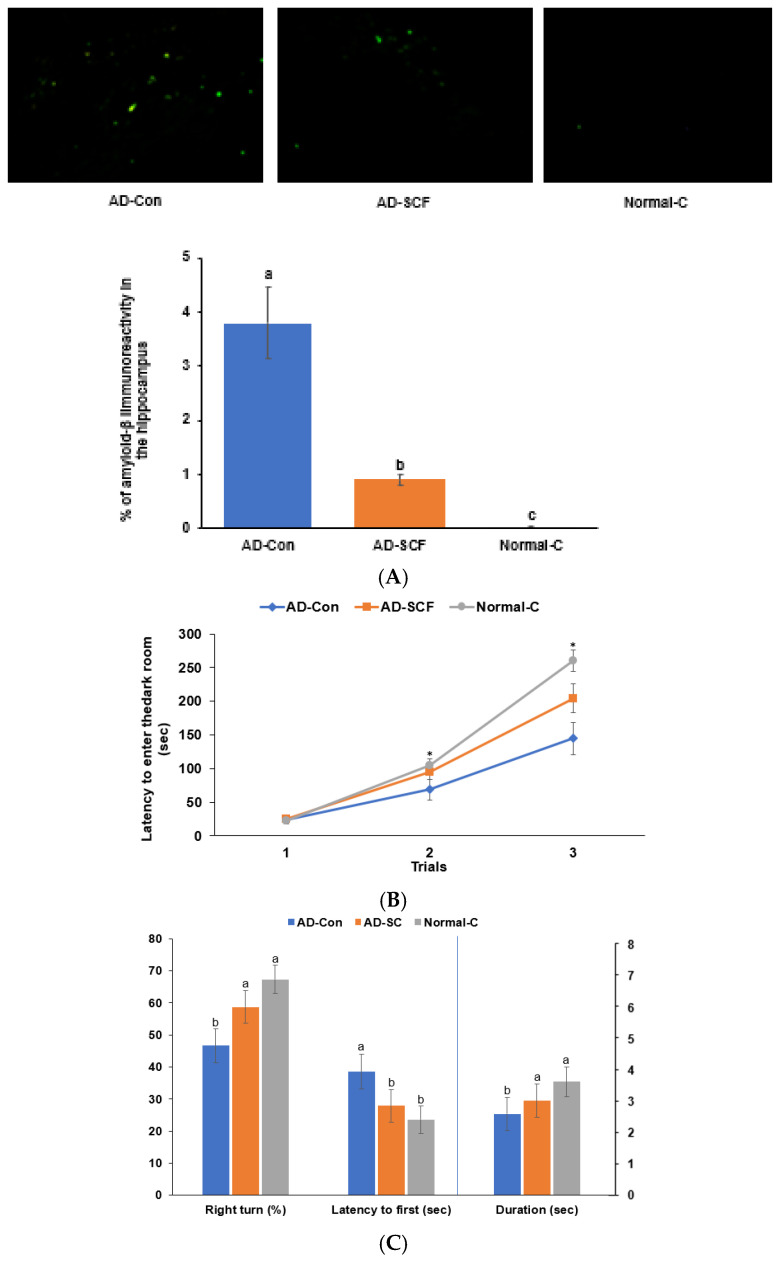
Amyloid-β (Aβ) staining in the hippocampus and memory function. (**A**). Aβ staining (n = 6). The green dot indicates Aβ deposition. (**B**). Latency time to enter the dark room in the passive avoidance test (n = 10). (**C**). Right turn percentage in the Y-maze test, latency to locate the zone with the platform, and the time spent in the platform zone on day 5 during the water maze test (n = 10). Each bar represents means ± standard deviation. At the end of the experimental period, the brain section (30 μm) was stained for Aβ using immunohistochemical techniques. The Aβ-positive cells were counted in the hippocampus area. The Aβ (25-35)-infused rats were fed high-fat diets with 0.5% dextrin (AD-Con) and 0.5% *Schisandra chinensis* Fructus water extract (SCF; AD-SCF) for 7 weeks. The Aβ (35-25)-infused rats were fed high-fat diets with 0.5% dextrin (Normal-C). * Significantly different among the groups in each trial at *p* < 0.05. ^a,b,c^ Different letters on the bar indicate significant differences between groups according to Tukey’s test (*p* < 0.05).

There was Aβ deposition in the hippocampus area in the AD-Con group, while there was no Aβ deposition in the hippocampus in the Normal-C group. The AD-SCF group showed reduced Aβ deposition compared to the AD-Con group (Figure 5A; *p* < 0.05).

Short-term memory impairment was evaluated using the passive avoidance and Y-maze tests, while spatial memory deficits were assessed through water maze tests (Figure 5B,C). During the passive avoidance test, the rats showed similar latencies in the first trial. After receiving an electric stimulation, the Normal-C and AD-SCF groups demonstrated slightly increased latencies compared to the AD-Con group in the second trial (*p* < 0.05; Figure 5B). In the third trial, the Normal-C and AD-SCF groups had greater latencies than the AD-Con group, with the Normal-C group displaying higher latencies than the AD-SCF group (Figure 5B). These results suggest that SCF improved the rats’ short-term memory compared to the AD-Con group, but not to the level of the Normal-C group. In the Y-maze tests, the AD-Con group exhibited a lower percentage of correct alternations than the Normal-C group. However, SCF intake (AD-SCF) significantly increased the percentage of correct alternations in comparison to the AD-Con group (*p* < 0.05; Figure 5C).

In the water maze test, the rats were tasked with finding the platform in zone 5 on two consecutive days, and on the fifth day, the platform was removed (*p* < 0.05; Figure 5C). The AD-Con group took longer to find zone 5 compared to both the Normal-C and AD-SCF groups, while the Normal-C and AD-SCF groups had similar latencies (Figure 5C). In the third trial, the AD-Con group spent less time in zone 5 searching for the platform compared to the Normal-C group, and the AD-SCF group had a duration that fell between the Normal-C and AD-Con groups (*p* < 0.05; Figure 4C).

#### 3.2.5. Markers of Neuroinflammation in the Blood and Hippocampus

The serum concentrations of the inflammatory markers TNF-α and IL-1β were much higher in the AD-Con group than in the Normal-C group, and the levels in the AD-SCF and Normal-C groups were similar (Table 3). Aβ accumulation in the hippocampus is linked to neuroinflammation and oxidative stress, and the parameters related to it were measured. The lipid peroxide contents in the hippocampus were higher in the AD-Con than in the Normal-C and AD-SCF groups (Table 3). Hippocampal AChE activity was also higher in the AD-Con than in the Normal-C and AD-SCF groups. The relative mRNA expressions of the markers of inflammation *TNF-*α, *IL-1β,* and *IL-6* were higher in the AD-Con group than in the Normal-C group, and the levels in the AD-SCF group were similar to those in the Normal-C group. The hippocampal *PTGS-2 (COX-2)* expression pattern was identical to *IL-1*β (Table 3). The TNF-α, IL-1β, and *PTGS-2* protein contents in the hippocampus showed similar patterns in terms of mRNA expression (Table 3). Therefore, Aβ deposition increased lipid peroxide and inflammatory cytokines in the hippocampus, creating a vicious cycle that increased Aβ deposition.

#### 3.2.6. BDNF and CTNF Expression in the Hippocampus

Memory function is closely linked to neuronal cell survival in the brain, particularly within the hippocampus. The mRNA expression levels of BDNF and CNTF—key growth factors for neuronal cells—were higher in the AD-Con group compared to the Normal-C group (Table 3). Although these levels were also elevated in the AD-SCF group compared to the AD-Con group, the increase in hippocampal BDNF expression in the AD-SCF group did not reach the levels observed in the Normal-C group. In contrast, CNTF expression in the AD-SCF group was elevated to the same level as that in the Normal-C group (Table 3). Tau protein expression in the hippocampus was higher in the AD-Con group than in the Normal-C group; however, SCF treatment did not reduce tau expression to the level of the Normal-C group (Table 3).

## 4. Discussion

Our study provides novel insights into the therapeutic potential of SCF in AD through an integrated approach combining a network pharmacology analysis and experimental validation. The network analysis revealed that SCF’s bioactive compounds, particularly gomisin A, gomisin R, and longikaurin A, target key AD-related pathways through AChE and PTGS2 modulation. These computational predictions were successfully validated in an Aβ-induced AD rat model, in which SCF administration significantly ameliorated multiple aspects of AD pathology. Notably, SCF demonstrated a unique multi-target therapeutic effect by simultaneously improving cholinergic function, reducing neuroinflammation, and normalizing glucose and lipid metabolism in the hippocampus. This comprehensive approach, addressing both cognitive and metabolic aspects of AD pathology, represents a significant advance over traditional single-target therapeutic strategies.

SCF contains various bioactive compounds including gomisin A, G, N, and R, schisandrin A, B, and C, and schizandra B, which have been reported to reduce oxidative stress, inflammation, fibrosis, and apoptosis and improve vasorelaxation, indicating its preventive and therapeutic potential for neurological diseases [22]. Our network pharmacology analysis identified several of these compounds in the SCF used in this study, particularly gomisin A and C and schisandrin A, B, and C, with a specific emphasis on the association between AChE activity and gomisin A and schizandra C. This analysis aligns with previous research that involved a thin-layer chromatography effect-directed analysis, which demonstrated that multiple SCF components, including 6-O-benzoylgomisin, deoxyschisandrin, gomisin A, gomisin G, schisandrin, schisandrin C, schisanhenol, schisantherin A, and schisantherin B, act as AChE inhibitors [23]. The predictions from our network pharmacology analysis were validated by our in vivo findings: the SCF treatment effectively decreased both lipid peroxide and pro-inflammatory cytokines in the hippocampus of Aβ (25-35)-infused rats and notably reduced AChE activity in the AD-SCF group [24].

The intake of SCF and its components mitigates memory impairment in memory-deficit-induced animals, such as those with an Aβ (1-42) infusion into the brain [25,26,27]. In the present study, Aβ (25-35) was bilaterally infused into the hippocampus, and its deposition was detected. Moreover, the rats in the AD-Con group showed short-term and spatial memory deficits measured by the Y-maze, passive avoidance, and water maze tests. Previous studies have revealed results consistent with those of the present study [25,26,28,29,30]. Deoxyschizandrin, schisantherin A, schisandrin A and C, and schisanhenol have been shown to alleviate memory impairment by inhibiting the production of inflammatory cytokines and lipid peroxides and improving insulin sensitivity in several animal models with memory deficits [25,26,28,29,30]. Furthermore, the intake of SCF and schisandrin C has been shown to decrease AChE in mice injected with Aβ (1-42) intracerebroventricularly [27,30], consistent with the results of the present study. In the present study, the Aβ (25-35) infusion in the hippocampus induced memory deficits which were mitigated with SCF intake, reducing AChE activity and neuroinflammation.

In our study, SCF demonstrated significant effects on both PTGS2 (COX-2) and AChE pathways, providing key insights into its therapeutic mechanism in AD. The modulation of PTGS2 (COX-2) and AChE by SCF components represents a significant finding in understanding SCF’s therapeutic potential in AD. Our study revealed that SCF’s effect on these pathways is particularly sophisticated, as it maintains a delicate balance in COX-2 signaling while effectively reducing AChE activity. This balanced modulation is crucial because while Aβ induces PTGS2 (COX-2) expression in hippocampal neurons as an early compensatory mechanism to prevent memory-related behavioral declines [31], the complete inhibition of COX-2 could be detrimental to normal brain function. This is similar to Ginkgo extract’s demonstrated ability to reduce memory impairment through COX-2/NF-κB signaling modulation [32,33]. The role of PTGS2 (COX-2) in memory function is complex. COX-2 knockout decreases *COX-2* expression, improving memory deficits [33]. SCF shows promise in regulating inflammatory pathways. However, SCF’s mechanism appears to be more nuanced, as it prevented the pathological increase in COX-2 expression seen in the AD-Con group while preserving the constitutive COX-2 expression necessary for the prostaglandin E2/cAMP pathway and the p-PKA-p-CREB-BDNF axis, which are essential for neuronal survival and memory function [34]. Our findings showed increased COX-2 expression in the AD-Con group, and SCF intake inhibited this increase without reducing hippocampal BDNF levels. These findings suggest that SCF may exert its reserve memory by modulating the COX-2-related signaling pathways in a balanced manner, maintaining COX-2’s homeostatic functions within the brain. Overall, the results of this study indicate that SCF could be a suitable preventive and therapeutic agent for AD-related memory deficits. However, further confirmation through human studies is still needed to establish the full clinical potential of this herbal medicine.

## 5. Conclusions

We explored the therapeutic potential of SCF in AD using an experimental rat model with amyloid-β-induced hippocampal infusion. Through a network pharmacology analysis, we identified significant intersections between SCF and AD-related targets, emphasizing critical connections such as gomisin A, gomisin C, schisandrin A, schisandrin B, and schisandrin C. Our experimental validation demonstrated SCF’s ability to mitigate the dysregulation in glucose and lipid metabolism, oxidative stress, neuroinflammation, and neuronal cell death associated with AD in rats injected with Aβ (25-35). Additionally, SCF modulated key genes such as *PTGS2* and *AChE,* which suggested it stimulated the parasympathetic nervous system to reduce neuroinflammation and oxidative stress. This highlights its potential therapeutic role in AD pathogenesis. Notably, we observed that the expression of memory-related factors such as *BDNF* and *CTNF* was preserved in the hippocampus, underscoring SCF’s neuroprotective effects. These findings suggest that SCF is a promising neuroprotective agent in AD treatment by stimulating the parasympathetic nervous system, warranting further investigations to elucidate its mechanisms of action. Moreover, the presence of bioactive compounds like gomisin A and schisandrin C in SCF, known for their inhibition of AChE activity, supports SCF’s potential preventive and therapeutic benefits in AD by stimulating the parasympathetic nervous system. Therefore, our study provides valuable insights into SCF’s potential as a candidate therapeutic agent for AD in a clinical setting. SCF is known to have oral bioavailability and multi-targets that could offer advantages over conventional single-target treatments. While these preclinical findings are promising, the standardization of SCF preparation and comprehensive human clinical trials are essential before its implementation in clinical practice, particularly focusing on dosage optimization and safety assessments for its long-term use.

## Figures and Tables

**Table 1:** Energy and glucose metabolism at the end of the experiment.

Variables	AD-Con	AD-SCF	Normal-C
Body weight (g)	304 ± 6.3	312 ± 11	313 ± 9.4
Weight gain for 5 weeks (g)	102 ± 8.3	109 ± 7.9	111 ± 9.7
Epididymal fat pads (g/kg bw)	3.5 ± 0.6 ^a^	2.8 ± 0.6 ^b^	2.7 ± 0.6 ^b^
Retroperitoneal fat/body weight (g/kg bw)	4.9 ± 0.8 ^a^	3.5 ± 0.6 ^b^	3.5 ± 0.5 ^b^
Visceral fat (g/kg bw)	8.4 ± 1.1 ^a^	6.2 ± 1.0 ^b^	6.1 ± 1.0 ^b^
Food intake (g)	19.4 ± 2.4	19.9 ± 2.5	19.3 ± 2.5
SC intake (mg/kg bw/day)	0 ± 0	199 ± 25	0 ± 0
Fasting serum glucose (mg/dL)	116 ± 7.66 ^a^	101 ± 5.66 ^b^	92.4 ± 4.23 ^c^
Fasting serum insulin (ng/mL)	1.22 ± 0.15 ^a^	1.12 ± 0.14 ^ab^	1.05 ± 0.11 ^b^
HOMA-IR	6.33 ± 0.75 ^a^	5.02 ± 0.62 ^b^	4.31 ± 0.58 ^c^

Values represent means ± standard deviation (n = 10). The amyloid-β (25−35)-infused AD model rats received the following assigned diets for 7 weeks: (1) a high-fat diet (43 energy % fat diet plus 0.5% dextrin) with ad libitum feeding (AD-Con); or (2) high-fat diet plus 0.5% *Schisandra chinensis* Fructus water extract (AD-SCF). The sham group rats received the same diet as those in the AD-Con (Normal-C) group for 7 weeks. ^a,b,c^ Different superscript letters within a column indicate significant differences between groups (*p* < 0.05).

**Table 2:** Lipid metabolism in serum and hippocampus.

Variables	AD-Con	AD-SCF	Normal-C
Serum triglyceride (mg/dL)	84.6 ± 7.6 ^a^	72.6 ± 7.5 ^b^	73.8 ± 6.9 ^b^
Serum total cholesterol (mg/dL)	122 ± 9.4 ^a^	117 ± 7.9 ^ab^	112.8 ± 6.9 ^b^
Serum TNF-α (pg/mL)	70.3 ± 7.1 ^a^	60.2 ± 6.5 ^b^	52.4 ± 5.3 ^c^
Serum IL-1β (pg/mL)	22.4 ± 2.3 ^a^	17.5 ± 2.0 ^b^	16.9 ± 1.9 ^b^
Serum lipid peroxide (MDA nmol/mL)	3.24 ± 0.35 ^a^	1.47 ± 0.25 ^b^	1.53 ± 0.27 ^b^
Hippocampal triglyceride (mg/g)	123 ± 14 ^a^	86.2 ± 10 ^b^	89.4 ± 9.6 ^b^
Hippocampal cholesterol (mg/g)	12.5 ± 1.8 ^a^	8.6 ± 1.0 ^b^	8.3 ± 0.9 ^b^
Hippocampal glycogen (mg/g)	6.9 ± 2.1 ^b^	13.6 ± 2.9 ^a^	12.7 ± 2.5 ^a^

Values represent means ± standard deviation (n = 10). TNF, tumor necrosis factor; IL, interleukin; MDA, malondialdehyde. The amyloid-β (25-35)-infused AD model rats received the following assigned diets for 7 weeks: (1) a high-fat diet (43 energy % fat diet plus 0.5% dextrin) with ad libitum feeding (AD-Con); or (2) high-fat diet plus 0.5% *Schisandra chinensis* Fructus water extract (AD-SCF). The sham group rats received the same diet as AD-Con (Normal-C). ^a,b,c^ Different superscript letters within a column indicate significant differences between groups according to Tukey’s test (*p* < 0.05).

**Table 3:** Neuroinflammation in the hippocampus.

Variables	AD-Con	AD-SCF	Normal-C
Lipid peroxides (MDA μmol/g)	0.44 ± 0.09 ^a^	0.29 ± 0.05 ^b^	0.32 ± 0.05 ^b^
Acetylcholinesterase activity (U/g protein)	0.19 ± 0.04 ^a^	0.08 ± 0.03 ^b^	0.09 ± 0.02 ^b^
TNF-*α* protein levels (pg/mL)	542 ± 44.3 ^a^	402 ± 36.3 ^b^	395 ± 33.2 ^b^
IL-1*β* protein levels (pg/mL)	175 ± 19.2 ^a^	131 ± 12.8 ^b^	139 ± 13.2 ^b^
PTGS-2 protein levels (pg/mL)	54.6 ± 4.5 ^a^	27.6 ± 3.5 ^b^	25.3 ± 2.3 ^b^
Relative mRNA expression of *TNF-α* (AU)	1.0 ± 0 ^a^	0.72 ± 0.10 ^b^	0.68 ± 0.08 ^b^
Relative mRNA expression of *IL-1β* (AU)	1.0 ± 0 ^a^	0.67 ± 0.08 ^b^	0.73 ± 0.09 ^b^
Relative mRNA expression of *IL-6* (AU)	1.0 ± 0 ^a^	0.75 ± 0.09 ^b^	0.71 ± 0.08 ^b^
Relative mRNA expression of *PTGS-2 (COX-2;* AU)	1.0 ± 0 ^a^	0.82 ± 0.08 ^b^	0.78 ± 0.08 ^b^
Relative mRNA expression of *BDNF* (AU)	1.0 ± 0 ^bc^	1.53 ± 0.21 ^b^	1.74 ± 0.19 ^a^
Relative mRNA expression of *CNTF* (AU)	1.0 ± 0 ^b^	1.42 ± 0.28 ^a^	1.36 ± 0.27 ^a^
Relative mRNA expression of *Tau* (AU)	1.0 ± 0 ^a^	0.78 ± 0.09 ^b^	0.57 ± 0.07 ^c^

Values represent means ± standard deviation (n = 10). The amyloid-β (25-35)-infused AD model rats received the following assigned diets for 7 weeks: (1) a high-fat diet (43 energy % fat diet plus 0.5% dextrin) with ad libitum feeding (AD-Con); or (2) high-fat diet plus 0.5% *Schisandra chinensis* Fructus water extract (AD-SCF). The sham group rats received the same diet as AD-Con (Normal-C) for 7 weeks. MDA, malondialdehyde; AU, arbitrary unit; TNF-α, tumor necrosis-α; IL, interleukin; PTGS-2, prostaglandin-endoperoxide synthase 2; COX-2, cyclooxygenase-2; BDNF, brain-derived neurotrophic factor; CNTF, ciliary neurotrophic factor. ^a,b,c^ Different superscript letters within a column indicate significant differences between groups according to Tukey’s test (*p* < 0.05).

## Data Availability

The original contributions presented in the study are included in the article/Supplementary Materials, further inquiries can be directed to the corresponding author.

## Supplementary Materials

The following supporting information can be downloaded at: https://www.mdpi.com/article/10.3390/nu16213751/s1, Figure S1. Venn diagram of *Schisandra chinensis* Fructus (SCF) bioactive compounds and Alzheimer’s disease targets. Figure S2. The cholinergic synapse mechanism involved in Alzheimer’s disease by *Schisandra chinensis* Fructus (SCF) in the KEGG. Figure S3. HPLC chromatogram of *Schisandra chinensis* Fructus. Table S1. Target Receptor and Enzyme Interactions of Schisandra chinensis Fructus components

## Abbreviations

SCF, *Schisandra chinensis* Fructus; AD, Alzheimer’s disease; *PTGS2,* prostaglandin-endoperoxide synthase-2; *AChE*, acetylcholinesterase; *APOE,* apolipoprotein E; *APP,* amyloid-β precursor protein; *PSEN,* presenilin; Aβ, amyloid-β; KEGG, Kyoto Encyclopedia of Genes and Genomes; GO, Gene Ontology; HOMA-IR, homeostasis model assessment for insulin resistance index; TNF-α, tumor necrosis-α; IL, interleukin; COX-2, cyclooxygenase-2; SD, standard deviations; ANOVA, analysis of variance; OB, oral bioavailability; DL, drug-likeness; *PPI, Protein–protein interaction; NR3C2,* nuclear receptor subfamily 3 group c member 2; *ESR1,* estrogen receptor alpha; *AR,* androgen receptor; *CHRM1,* cholinergic receptor muscarinic 1; CHAT, choline O-acetyltransferase; AUC, area under the curve; OGTT, oral glucose tolerance test; PKA, protein kinase A; CREB, cAMP-response element binding protein.
