# Supplementary material for: Efficacy and Mechanism of Schisandra chinensis Fructus Water Extract in Alzheimer’s Disease: Insights from Network Pharmacology and Validation in an Amyloid-β Infused Animal Model
Source: Nutrients. 2024 Oct 31;16(21):3751. doi: 10.3390/nu16213751 (PMC11547720; doi:10.3390/nu16213751)
Supplement: Supplementary file 1 [file nutrients-16-03751-s001.zip › nutrients-3279609-supplementary.pdf]

Supplementary figures

Figure S1. Venn diagram of *Schisandra chinensis* Fructus (SCF) bioactive compounds and Alzheimer’s disease targets

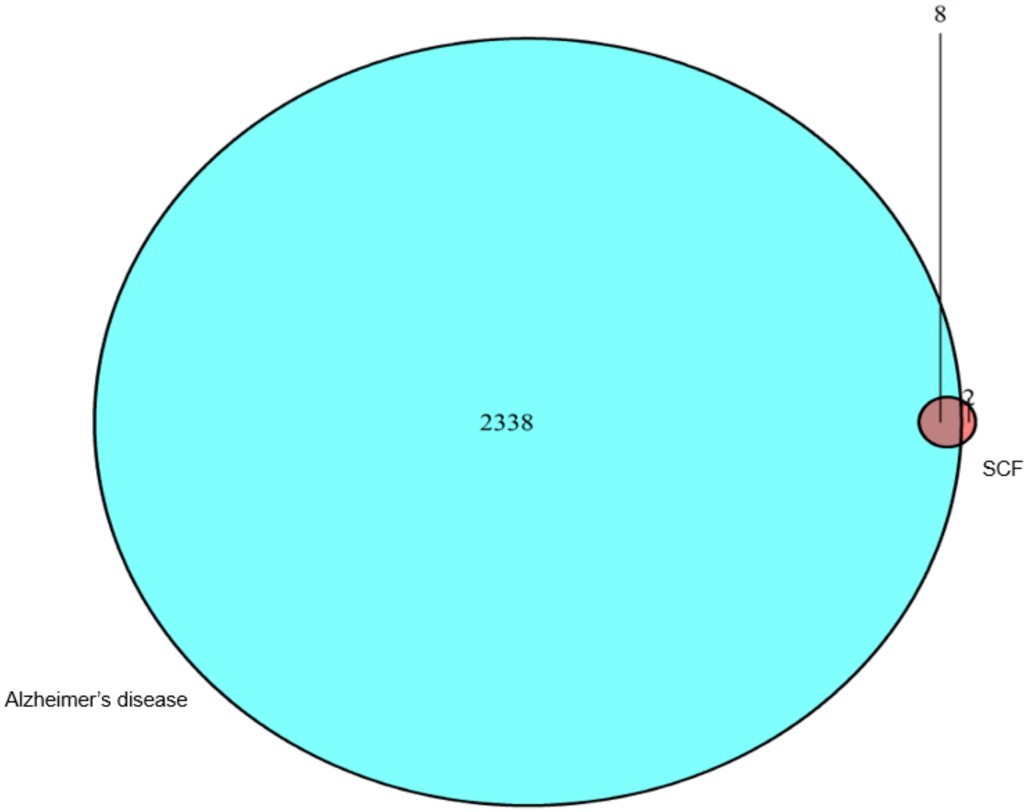

Figure S2. Kyoto Encyclopedia of Genes and Genomes (KEGG) enrichment analysis and the cholinergic synapse mechanism involved in Alzheimer's disease by *Schisandra chinensis* Fructus (SCF) in the KEGG

A. KEGG enrichment analysis

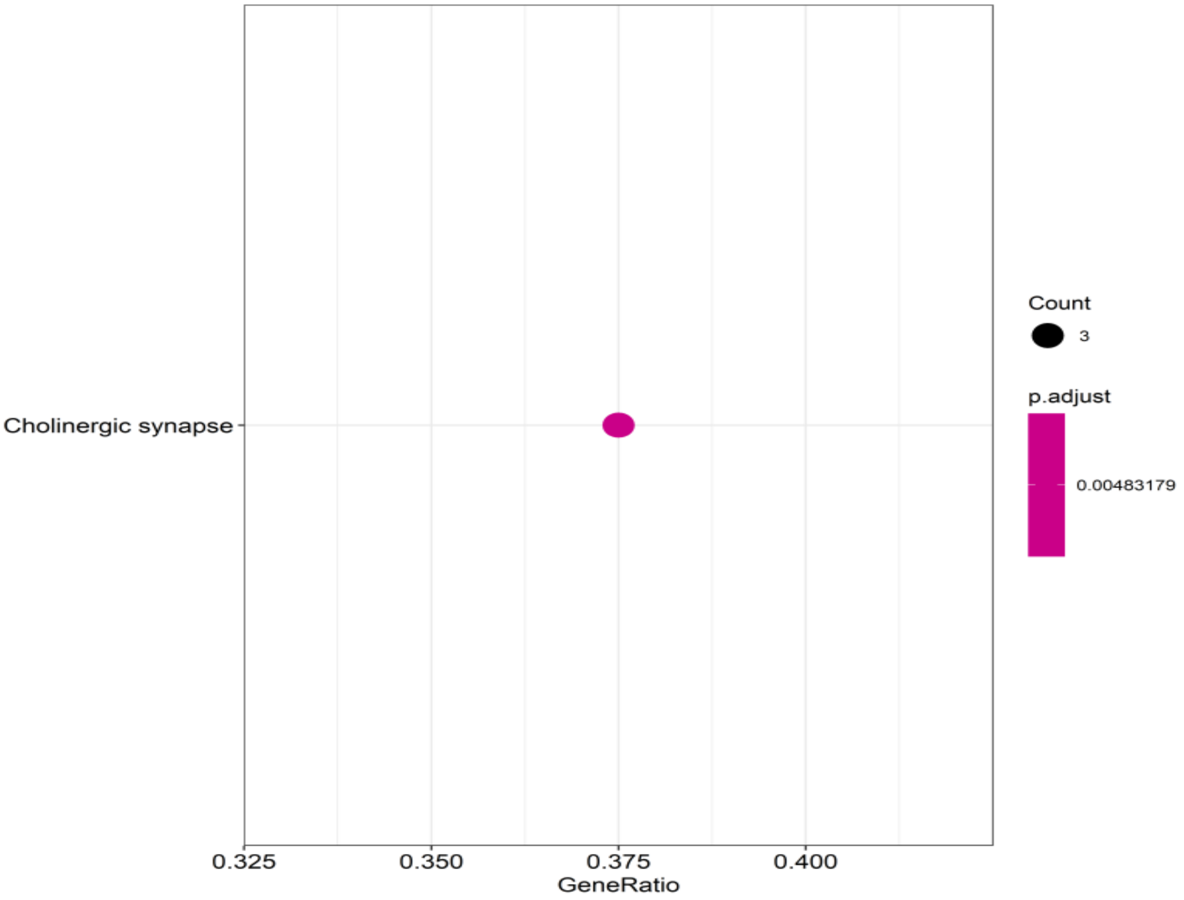

B. The cholinergic synapse mechanism involved in Alzheimer's disease by *Schisandra chinensis* Fructus (SCF) in the KEGG

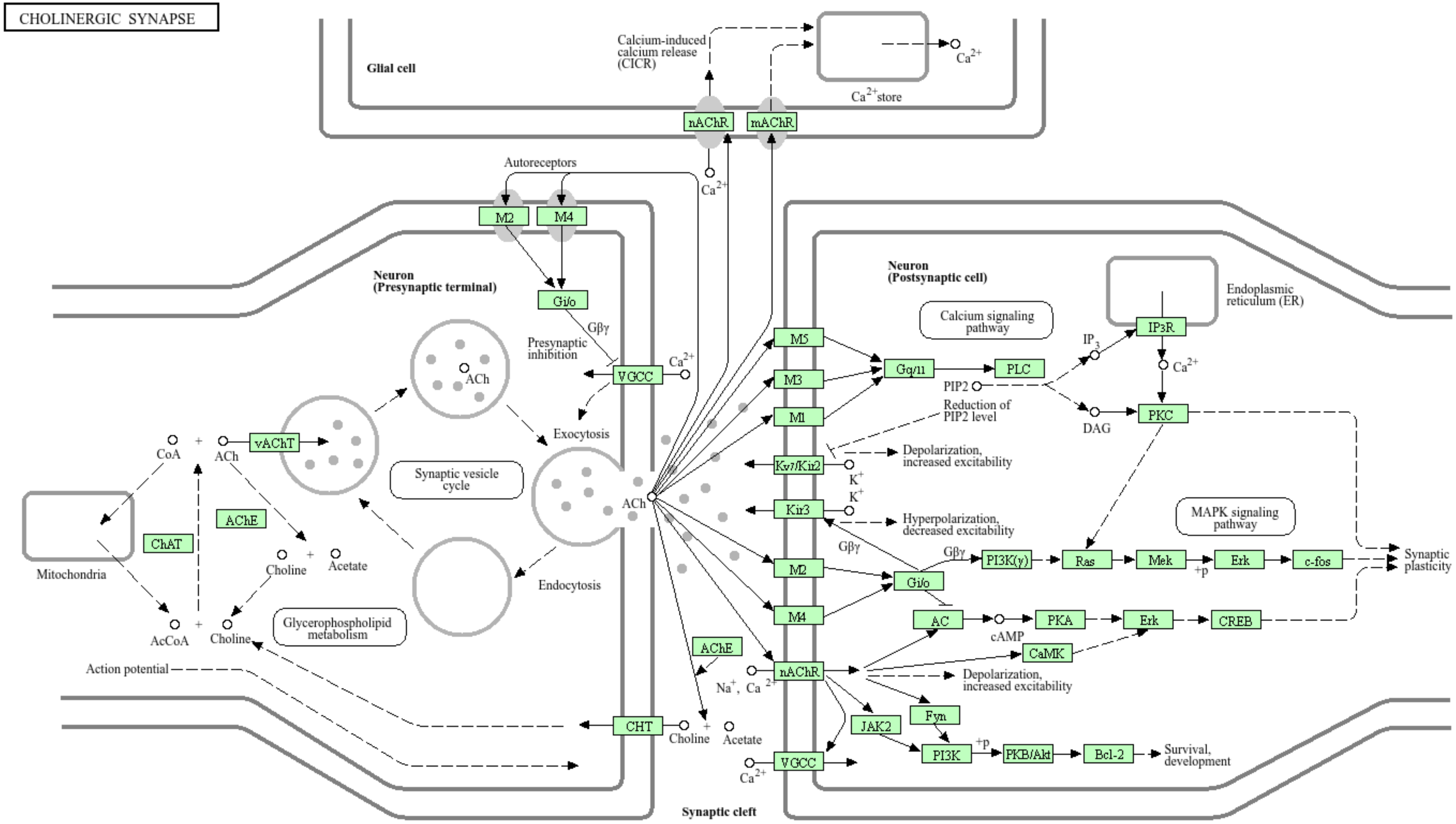

Figure S3. HPLC chromatogram of *Schisandra chinensis* Fructus

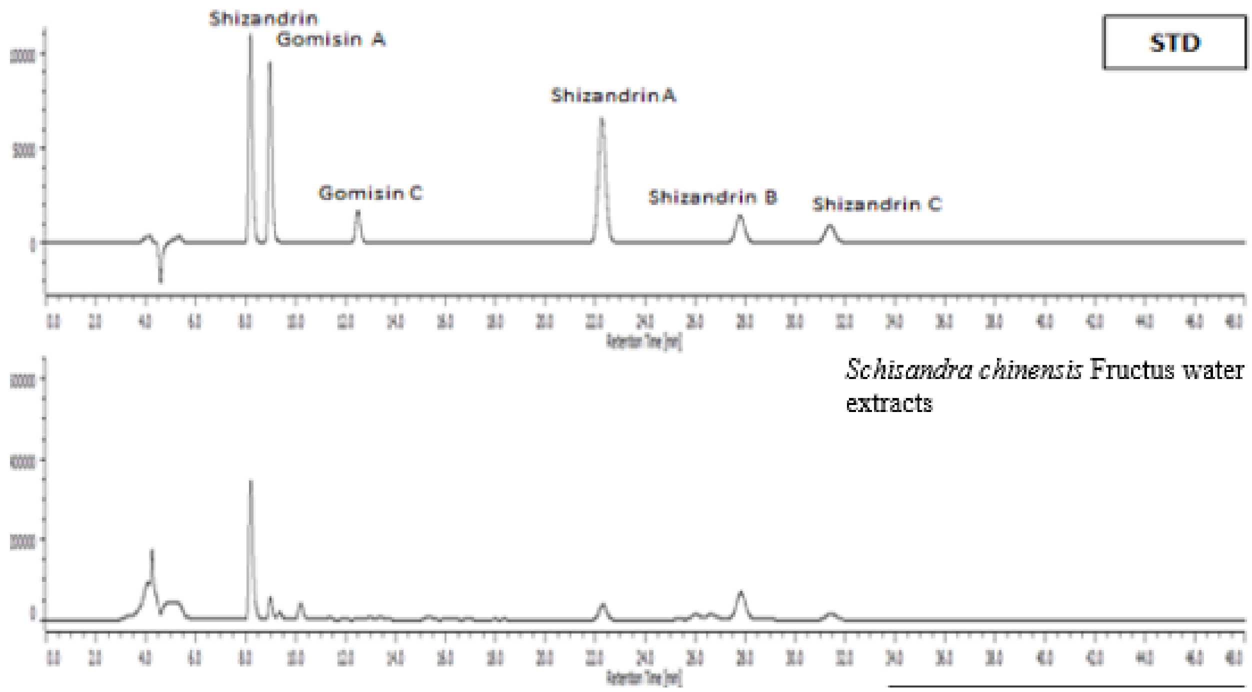

Table S1. Target Receptor and Enzyme Interactions of *Schisandra chinensis* Fructus components

Molecular ID	Molecular Name	Target
MOL004624	Longikaurin A	Muscarinic acetylcholine receptor M1
MOL004624	Longikaurin A	Gamma-aminobutyric-acid receptor alpha-2 subunit
MOL004624	Longikaurin A	Gamma-aminobutyric-acid receptor alpha-3 subunit
MOL004624	Longikaurin A	Muscarinic acetylcholine receptor M2
MOL004624	Longikaurin A	Trypsin-1
MOL004624	Longikaurin A	Gamma-aminobutyric-acid receptor subunit alpha-6
MOL005317	Deoxyharringtonine	Androgen receptor
MOL005317	Deoxyharringtonine	Mineralocorticoid receptor
MOL008956	Angeloylgomisin O	Androgen receptor
MOL008956	Angeloylgomisin O	Coagulation factor Xa
MOL008956	Angeloylgomisin O	DNA topoisomerase II
MOL008957	Schizandrer B	Prostaglandin G/H synthase 2
MOL008957	Schizandrer B	DNA topoisomerase II
MOL008968	Gomisin-A	Thrombin
MOL008968	Gomisin-A	Nitric-oxide synthase, endothelial
MOL008968	Gomisin-A	Acetylcholinesterase
MOL008974	Gomisin G	DNA topoisomerase II
MOL008978	Gomisin R	Estrogen receptor
MOL008978	Gomisin R	Androgen receptor
MOL008978	Gomisin R	Coagulation factor Xa
MOL008978	Gomisin R	Prostaglandin G/H synthase 2
MOL008978	Gomisin R	DNA topoisomerase II
MOL008978	Gomisin R	Trypsin-1
MOL008978	Gomisin R	Proto-oncogene serine/threonine-protein kinase Pim-1
MOL008978	Gomisin R	Nuclear receptor coactivator 2
MOL008978	Gomisin R	Calcium-activated potassium channel subunit alpha 1
MOL008992	Wuweizisu C	Acetylcholinesterase
MOL008992	Wuweizisu C	Gamma-aminobutyric acid receptor subunit alpha-1
MOL008992	Wuweizisu C	Dipeptidyl peptidase IV
MOL008992	Wuweizisu C	Cytochrome P450-cam
